# Recent Advances in Wearable Biosensors for Non-Invasive Detection of Human Lactate

**DOI:** 10.3390/bios12121164

**Published:** 2022-12-13

**Authors:** Yutong Shen, Chengkun Liu, Haijun He, Mengdi Zhang, Hao Wang, Keyu Ji, Liang Wei, Xue Mao, Runjun Sun, Fenglei Zhou

**Affiliations:** 1 School of Textile Science and Engineering, Xi’an Polytechnic University, Xi’an 710048, China; 2 Key Laboratory of Functional Textile Material and Product of the Ministry of Education, Xi’an Polytechnic University, Xi’an 710048, China; 3 Shaanxi College Engineering Research Center of Functional Micro/Nano Textile Materials, Xi’an Polytechnic University, Xi’an 710048, China; 4 Engineering Research Center for Knitting Technology of the Ministry of Education, Jiangnan University, Wuxi 214122, China; 5 Centre for Medical Image Computing, Department of Medical Physics and Biomedical Engineering, University College London, London WC1E 6BT, UK

**Keywords:** lactate detection, non-invasive, wearable biosensors, flexible substrates

## Abstract

Lactate, a crucial product of the anaerobic metabolism of carbohydrates in the human body, is of enormous significance in the diagnosis and treatment of diseases and scientific exercise management. The level of lactate in the bio-fluid is a crucial health indicator because it is related to diseases, such as hypoxia, metabolic disorders, renal failure, heart failure, and respiratory failure. For critically ill patients and those who need to regularly control lactate levels, it is vital to develop a non-invasive wearable sensor to detect lactate levels in matrices other than blood. Due to its high sensitivity, high selectivity, low detection limit, simplicity of use, and ability to identify target molecules in the presence of interfering chemicals, biosensing is a potential analytical approach for lactate detection that has received increasing attention. Various types of wearable lactate biosensors are reviewed in this paper, along with their preparation, key properties, and commonly used flexible substrate materials including polydimethylsiloxane (PDMS), polyethylene terephthalate (PET), paper, and textiles. Key performance indicators, including sensitivity, linear detection range, and detection limit, are also compared. The challenges for future development are also summarized, along with some recommendations for the future development of lactate biosensors.

## 1. Introduction

Lactate is one of the significant metabolites of the anaerobic phase of glycolysis and is significant in clinical diagnostics, sports medicine, and food analysis [1]. Determining lactate concentration in physiological fluids is crucial for evaluating patient situations because it is linked to a number of disorders, including shock, respiratory failure, heart failure, and drug or toxin consumption. It is also helpful in sports medicine for assessing the physical status of athletes who have been engaged in strenuous sports for a long time [2].

Traditional lactate detection equipment is often used to detect the content of lactate in blood [3,4]. In order to understand lactate levels in critical care patients and persons who have difficulty in blood sampling and need to regularly monitor lactate levels (such as diabetes patients and athletes), repeated blood collection must be performed frequently. It is not only invasive and inconvenient, but also increases the risk of infection. Therefore, non-invasive detection of lactate content in matrices other than blood is extremely necessary [5]. It was found that the lactate content in human body fluid and blood had a certain corresponding relationship. For instance, the concentration of lactate in human blood at rest was 0.5–2.2 mM, and that in human sweat was 4–25 mM [6]. During intensive activities, as muscle cells switched to anaerobic metabolism, lactate concentration in blood could rise to 30 mM or higher, and that in sweat also increased to more than 50 mM [7]. Therefore, the direct detection of lactate content in body fluid could replace blood detection to a certain extent and become an excellent detection method [8,9,10,11].

Because of their straightforward design, simple operation, low cost, ease of miniaturization, high sensitivity, good selectivity, and quick real-time monitoring, biosensors have been favored by researchers as an emerging high-tech detection method and widely used in medical testing, clinical diagnosis, environmental testing, food analysis, and other fields [12,13]. To ensure good contact between the sensor and the skin, it was typically implemented by applying the sensor directly to the skin (such as electronic skin or screen-printed tattoo stickers), integrating the sensor into a wristband and medical applicator, or sewing it into clothing [14]. 

**Figure 1:**
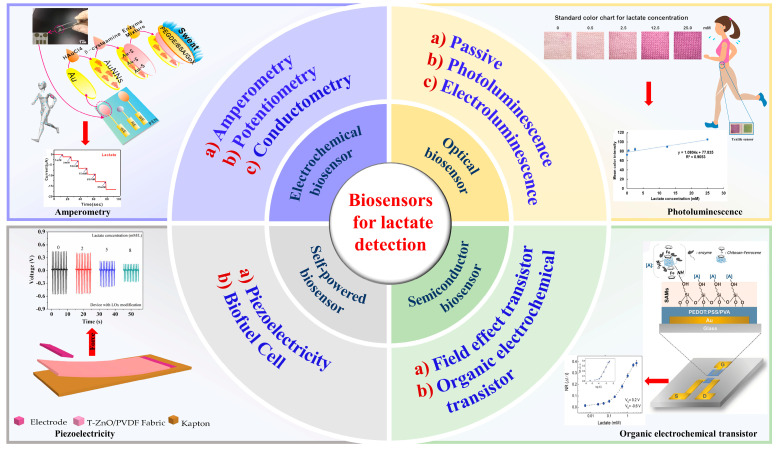
Biosensors for lactate detection in human biofluid. Reprinted with permission from ref. [16]. Copyright 2021 Elsevier. Reprinted with permission from ref. [17]. Copyright 2019 Elsevier. Reprinted with permission from ref. [18]. Copyright 2016 WILEY-VCH. Reprinted with permission from ref. [19]. Copyright 2020 MDPI.

The structure of the biosensor consists of two main parts: the recognition element and the conversion element. The recognition element, also referred to as the sensitive element, is the essential component of a biosensor device that can detect and respond to a measured variable with a high sensitivity and output a physical quantity that has a specified relationship with the measured variable [15]. The selectivity of the recognition element allows the sensor to selectively respond to one or a certain type of analyte, thus avoiding the mutual interference with other substances. The recognition element is a biological molecule (including enzymes, antibodies, and nucleic acids) in nature. The conversion element, also called transducer, can transform the data generated from the recognition element into a readable signal. When the molecular recognition element interacts with the recognized object, its physical and chemical parameters will change, and then these parameters will be converted into qualitative or quantitative electrical signals or optical signals related to the characteristics of the analyte through the transducer. There are currently several different types of wearable biosensors available for determining the amount of lactate in body fluids, including electrochemical, optical, semiconductor, and self-powered biosensors, as illustrated in Figure 1 [16,17,18,19].

For the stable and accurate detection of lactate, several properties of the biosensors must be optimized, such as sensitivity, detection limit, selectivity, and response time. Sensitivity refers to the severity of changes in the concentration of analyte detected by the sensor. Since the regulation of human lactate during secreting process keeps its concentration within a small fluctuation range, highly sensitive sensors are required to capture the small physiological related concentration fluctuations [20]. In order to improve the sensitivity of the sensor, researchers applied metal oxides [21,22,23,24,25,26], carbon nanotubes (CNTs) [27], graphene [28,29], novel two-dimensional materials (e.g., MXene) [30,31]. and porous materials (e.g., MOFs) [32,33,34] to various types of lactate biosensors. The majority of these materials are nanomaterials because of their high specific surface area and good biocompatibility [35,36,37]. The detection limit of biosensors referring to the lowest analyte concentration that can be detected or identified is also lowered by the addition of nanomaterials [29]. The ability of a sensor to selectively detect a target analyte in the presence of other possibly interfering substances during detection is referred to as selectivity. Enzymes (lactate dehydrogenase (LDH) and lactate oxidase (LOx/LOD)) are frequently added to make the sensor selective. Prussian blue (PB), which is a good electron transfer mediator and can lower the redox reaction potential, can also be employed to further limit the introduction of interfering signals and improve sensor selectivity [38,39,40,41,42,43]. Additionally, a layer of semi-permeable membrane, made by perfluorosulfonic acid type polymers (Nafion) with specific size or charge molecule repulsion can also be coated on the electrode surface to lessen the passage of interfering species [9,44]. Response time is the time required for the sensor to respond and stabilize to a reliable value when the analyte concentration changes. A fast response is important for continuous dynamic monitoring to ensure that changes in lactate concentration are captured in real time. When the recognition element of the sensor has a higher activity or a thinner electrode modification film, a faster response time can be obtained [45]. 

In this paper, the main types and preparation process of wearable lactate biosensors are briefly introduced, and several key properties of the biosensors, such as sensitivity, detection limit, and linear detection range, are compared. The substrate materials used for sensor manufacturing and sample collection are presented. The benefits and drawbacks of enzymatic and non-enzymatic wearable lactate biosensors are summarized, and the development prospect of flexible wearable biosensors in the future is prospected.

## 2. Electrochemical Biosensors

Electrochemical biosensors are of increasing interest to researchers and one of the most mature and widely used sensors at present. Electrochemical biosensors can convert the concentration of the target compound and biologically active substances into electrical signals (such as current, potential, impedance, and conductance). Because of their ability to provide shorter, more accurate, and more sensitive responses in a cost-effective manner, they can also be combined with microfluidic systems to develop miniaturized components on a single platform.

### 2.1. Types of Electrochemical Biosensors

**Figure 2:**
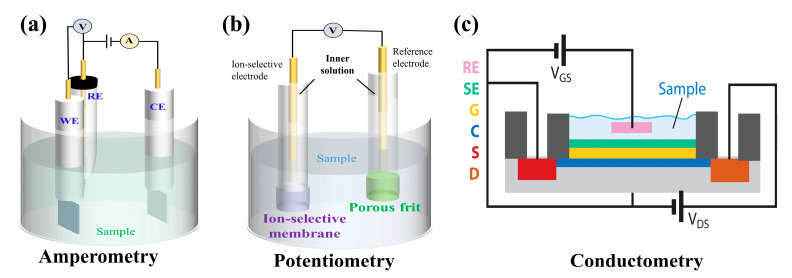
Schematic of the working principles for different types of electrochemical biosensors: (**a**) Amperometry (WE: Work Electrode, RE: Reference Electrode, CE: Counter Electrode), (**b**) Potentiometry, (**c**) Conductometry. Reprinted with permission from ref. [46]. Copyright 2019 Annual Reviews.

Electrochemical biosensors can be categorized in two ways: First, they can be divided into amperometric, potentiometric, conductive/impedance biosensors based on their output signal and working mode. Second, they can be classified as enzyme, nucleic acid aptamer, immune, and microbial sensors according to the type of biological small molecules or biological related substances they can detect. These different kinds of electrochemical biosensors are described in detail below, and the working principles of different types are shown in Figure 2 [46].

#### 2.1.1. Amperometric Lactate Biosensor

The amperometric lactate biosensor detects the current by applying a specific voltage between the working electrode and the reference electrode, and the measured current is proportional to the concentration of analyte in the solution for quantitative analysis [21,47]. Compared with other types of electrochemical biosensors, the amperometric type can simultaneously measure multiple substances and reflect the content of the analyte more intuitively and has the advantage of being disposable.

In the preparation of amperometric electrochemical biosensors, LOx or LDH was frequently immobilized on the electrode surface of the sensor [48]. One study used PB and LOx to make an amperometric biosensor for the detection of lactate in saliva with the detection range of 0.025–0.25 mM, the detection limit of 0.01 mM, and a good linearity (*r^2^ = 0.999*) [9]. To improve the sensitivity of the biosensor, a dual-enzyme amperometric biosensor could also be prepared by adding horseradish peroxidase (HRP) to LOx for the detection of lactate in saliva. The biosensor demonstrated a good linearity at lactate concentrations between 0.1 and 1.0 mM in 0.1 M phosphate buffer (pH 7.0), a high sensitivity in the linear dynamic range, and a detection limit of 0.013 mM [49]. 

#### 2.1.2. Potentiometric Lactate Biosensor

The potentiometric lactate biosensor is to use the electric potential generated by the action of the measured substance dissolved in the electrolyte solution on the electrode as the output of the sensor. The potentiometric type measuring instrument has been favored by people over the past few decades since it uses a low-cost detection method and occupies small space.

However, the selectivity of the potentiometric type is relatively low, and due to some limitations of the enzyme-based potentiometric sensor, such as the poor chemical and thermal stability, a decrease occurred in the performance of the sensor after a long-term operation. Therefore, more research has been focused on non-enzymatic potentiometric sensors. For example, electrodes modified with polypyrrole (PPy) films allowed for the effective fabrication of this type of biosensor, which demonstrated an excellent linearity in the range of 0.1 to 10.0 mmol L^−1^, and a detection limit of 81 mol L^−1^ [50]. It was also possible to employ a screen-printed carbon electrode (SPCE) modified with a stable Fe^3+^ solution to detect lactate with the linear response range of 1–180 mM [51]. To further enhance the performance of the sensor, a potentiometric biosensor based on a flexible array of silver paste electrodes and copper-doped zinc oxide (ZnO) sensing membranes modified by iron-platinum (Pt) nanoparticles (NPs) was fabricated for the detection of lactate in the human body with the detection range of 0.2–5 mM and sensitivity of 25.32 mV mM^−1^ [52].

#### 2.1.3. Conductive/Impedance Lactate Biosensor

The change in ion concentration by the oxidation and reduction of chemicals in the solution can lead to a change in the signal of the resistance or impedance of the electrolyte, which is the detection principle of the conductivity and impedance types. They have the characteristics of simple electrode configuration and miniaturization, while being seldom used due to the fewer carriers affecting the conductivity process [53]. 

For the detection of lactate in sweat, researchers created a highly sensitive and selective impedance biosensor. A cross-linking agent was used to embed LOx in the nanopore and immobilize it in the active sensing region of nanostructured ZnO. A change in impedance was caused by the catalytic oxidation of lactate to pyruvate and H_2_O_2_, which made it possible to detect lactate in sweat. Electrochemical impedance spectroscopy (EIS) monitoring revealed that the lactate biosensor had a dynamic detection range of 1–100 mM and a detection limit of 1 mM in human sweat. The sensor stability studies showed that the lactate biosensing response would decrease about 30% over a four days when stored at 4°C [54]. LOx and graphene oxide nanosheets were also used to develop an impedance-based biosensor that enabled continuous, non-invasive, real-time monitoring of lactate in human sweat. The resultant sensor achieved a detection range of 1–100 mM, a detection limit of 1 mM, and a correlation of 0.955 between the sensor response and the real lactate concentration (1.324–113.4 mM) [47]. 

### 2.2. Preparation of Electrochemical Biosensors

#### 2.2.1. Screen Printing

**Figure 3:**
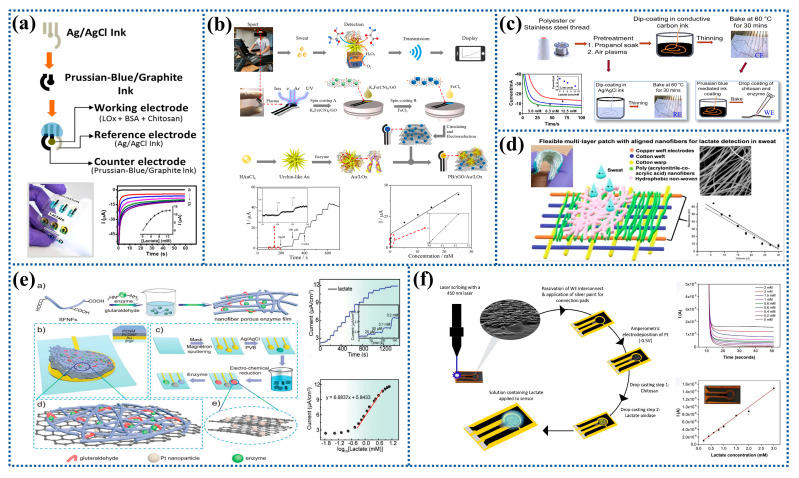
Preparation process and characterization of electrochemical biosensors for lactate detection: (**a**) A fully integrated wireless eyeglasses-based biosensor platform for monitoring lactate in sweat in real time. Reprinted with permission from ref. [40]. Copyright 2017 Royal Society of Chemistry. (**b**) A wearable lactate biosensor fabricated by in-situ preparation of PB sensing membrane incorporated with rGO and urchin-like Au NPs on flexible SPCE. Reprinted with permission from ref. [65]. Copyright 2022 Elsevier. (**c**) Electrochemical sensor for detecting lactate using the wires as substrate. Reprinted with permission from ref. [39]. Copyright 2020 Nature Publishing Group. (**d**) Electrochemical biosensor prepared by electrospinning for detecting lactate in human sweat. Reprinted with permission from ref. [53]. Copyright 2021 Elsevier. (**e**) Coupling of silk fibroin nanofibrils enzymatic membrane with ultra-thin PtNPs/Graphene film to acquire long and stable on-skin sweat lactate sensing. Reprinted with permission from ref. [72]. Copyright 2021 Wiley-VCH. (**f**) Electrochemical sensor for enzymatic lactate detection based on laser-scribed graphitic carbon. Reprinted with permission from ref. [73]. Copyright 2022 Elsevier.

Screen printing is a new low-cost printing technology and has shown promise in the production of electrochemical biosensors. The advantage of the screen-printing electrode (SPE) over traditional rod electrodes lies in their integration into a variety of portable test equipment due to its small size. To increase the selectivity and anti-interference of the sensor, enzymes, carbon materials, metals, polymers, electrochemical mediators, and complexing agents have been frequently employed to modify electrodes [14,55,56,57,58]. For instance, the Ag/AgCl conductive ink was printed to prepare the reference electrode, and the PB graphite ink was printed to prepare the working electrode and the counter electrode by screen printing. The results showed that the lactate biosensor had a linear detection range of 0–8 mM with the detection limit of 0.39 mM, as shown in Figure 3a [40].

The SPCE is frequently modified using metal NPs, polyaniline (PANI), graphene, and other two-dimensional materials to prepare wearable electrochemical biosensors [30,59,60,61,62,63]. For instance, the graphene-modified electrode used in an amperometric lactate biosensor had a distinct redox peak with a lower potential separation peak than the unmodified electrode, showing an enhanced electron transfer rate [64]. Other researchers prepared a wearable lactate electrochemical biosensor for real-time monitoring of epidermal sweat lactate. As shown in Figure 3b, sensing membranes incorporating rGO/PB and urchin-like Au NPs were fabricated in-situ on flexible SPCE. The results showed that the lactate sensor had a sensitivity of 40.6 μA mM^−1^ cm^−2^ in the concentration range of 1–222 μM and a sensitivity of 1.9 μA mM^−1^ cm^−2^ in the concentration range of 0.222–25 mM [65].

Additionally, the SPCE can also be modified by using molecularly imprinted polymers (MIPs) to detect human lactate in sweat [66]. A wearable electrochemical biosensor based on MIPs and silver nanowires (Ag NWs) was proposed by one research group. It consisted of a carbon working electrode coated with MIPs-Ag NWs, an Ag/AgCl reference electrode, and a carbon counter electrode for the non-invasive monitoring of lactate in human sweat. It was found that high sensitivity and specificity for the detection of lactate in the range of 10^−6^ M–0.1 M with the detection limit of 0.22 μM could be achieved [67].

#### 2.2.2. Drop Coating

The drop coating method is to drop the polymer or nanomaterial on the surface of the electrode. The modified coating film is combined with the electrode to complete the modification of the electrode when the solvent is evaporated and dried. Generally, electrochemical biosensors for lactate detection often consist of a modified glassy carbon electrode (GCE). For instance, exfoliated molybdenum disulfide (MoS_2_) nanosheets can be created and subsequently dropped onto the GCE surface together with LOx to prepare modified GCE, which demonstrated a linear detection range of 0.056–0.77 mM, a sensitivity of 6.2 μA mM^−1^, and a detection limit of 17 μM for lactate measurement [37].

Transition metal oxides have been widely used in non-enzymatic biosensors due to their good electrochemical catalytic properties and stability. As an illustration, the cobalt oxide nanomaterial was used to modify GCE by the drop-coating method. The sensor had a detection limit of 0.006 mM and varied linearly in the range of 0.05–3 mM [21]. Porous mesoporous nickel oxide (NiO) obtained by the inverse micelle sol-gel process was dispersed in Nafion, and the NiO/Nafion nano-dispersion was drop-coated onto GCE as the working electrode to prepare the lactate biosensor. The results showed that the mesoporous NiO-based biosensor had a sensitivity of 62.35 μA mM^−1^ cm^−2^, a detection limit of 27 μM, and a detection range of 0.01–27.6 mM [23]. Similarly, a mesoporous copper oxide (CuO) could also be prepared by the inverse micelle sol-gel method and then titrated onto GCE to prepare a lactate biosensor. The sensitivity of this mesoporous CuO-based biosensor was 80.33 μA mM^−1^ [22].

Typically, the drop-coating technique is also used to create fabric-based wearable biosensors. For instance, carbon was coated on nylon wires as working and counter electrodes, and Ag/AgCl was coated on nylon wire as reference electrode. The prepared yarn electrodes were braided to prepare a wearable electrochemical lactate sensor. The results showed that it had a little effect on the output signal when the deformation occurred, indicating that the biosensor had a good robustness [68]. The polyethylene (PE) wire can also be coated with carbon ink to act as a counter electrode. PB and LOx solution was dripped on the surface of the PE/Carbon wire to create the working electrode. The Ag/AgCl/PE wire was dipped in the PVB solution to prepare the reference electrode. As shown in Figure 3c, the constructed wire sensor array was integrated into a patch for the detection of lactate in sweat with the sensitivity of 900 nA mM^−1^ [39].

#### 2.2.3. Others

In addition to screen printing and drop coating, electrospinning, magnetron sputtering, direct laser writing, and inkjet printing techniques have also been used to make electrodes of electrochemical biosensor [25,69,70]. Due to the large specific surface area of electrospun nanofibers, optimal contact can be achieved between target molecules (biomarkers) and biosensors to significantly improve the sensitivity, selectivity, and detection limit of biosensors [71]. Some scholars used electrospun oriented poly (acrylonitrile-co-acrylic acid) (P(AN-co-AA)) nanofibers to prepare electrochemical biosensors. The electrical resistance changed with lactate content when artificial sweat was put to the nanofibers. As can be seen in Figure 3d, the linear correlation coefficient was 0.94 in the 27–270 ppm range [53].

It was also possible to sputter a layer of gold on the substrate by magnetron sputtering and subsequently cover with one-pot reaction-produced PtNPs-loaded graphene (Pt-G) nanocomposites as the electrode of the sensor. Afterwards, LOx and silk fibroin nanofibers (SFNFs) were gradually drop-coated on the Pt-G membrane, and glutaraldehyde was then added to form cross-links to prepare a sensor for monitoring the concentration of lactate in sweat. Figure 3e depicts a linear detection range of 0.4–6 mM and a sensitivity of 6.68 μA cm^−2^ [log_10_ (mM)]^−1^ for the sensor. It was possible to use this bioactive porous enzyme nanofiber membrane composed of SFNFs and enzymes to facilitate electron transfer between the electrodes and the enzymes [72].

Some researchers used the laser direct writing method to make a laser-scribed graphitic (LSG) carbon electrode on a polyimide (PI) substrate, then deposited Pt on the electrode, and finally poured chitosan (CS) and LOx on the electrode to prepare a flexible laser-engraved graphite carbon based lactate biosensors, which could detect lactate linearly in the range of 0.2–3 mM (*R^2^* > *0.99*) with the detection limit of 0.11 mM and the sensitivity of 35.8 µA mM^−1^ cm^−2^, as shown in Figure 3f [73]. Another research group fabricated the working electrode for a lactate sensor consisting of a mediating layer, which was made by drop-casting tetrathiafulvalene (TTF)/CNT suspension on the gold electrode, followed by LOx immobilized in the CS/CNT suspension. The effect of polyvinyl chloride (PVC) membrane on the sensor performance was also compared. The sensitivity of the working electrode with and without the membrane was 3.28 ± 8 μA mM^−1^ and 0.43 ± 0.11 μA mM^−1^, and the linear range was 0–20 mM and 0–30 mM. It was shown that the covering PVC film reduced the transport rate of lactate to the electrode [45].

In Table 1, different types of electrochemical biosensors are compared primarily in terms of materials used to make the working electrodes, detection methods, substances used for the detection, and other sensor-specific characteristics. Through comparison, it is found that the sensitivity and detection range of electrochemical biosensors can be significantly improved by adding nanomaterials, such as metal oxides and carbon materials. However, it is worth noting that the performance of electrochemical biosensors is influence by a variety of factors, such as storage time and environment, test equipment and conditions, and device preparation methods. Therefore, researchers began to study non-enzymatic electrochemical biosensors, which avoided the requirements storage conditions and time with respect to enzymes, allowing the biosensor to maintain good performance after a long storage time.

## 3. Optical Biosensors

Optical biosensors use optical signals to detect materials of interest, and generally consist of a sensing layer, an optical signal conversion device, and an amplification processing device. Due to their non-destructive mode of operation and high signal generation and rapid reading speed, optical biosensors are the most commonly used biosensors [85]. 

### 3.1. Types of Optical Biosensors

**Figure 4:**
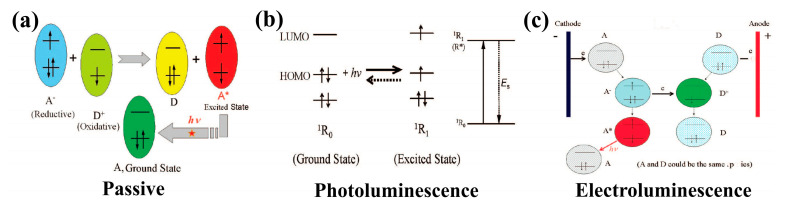
Schematic of the working principles for different types of optical biosensors: (**a**) Passive, (**b**) Photoluminescence (R*: the excited state species), (**c**) Electroluminescence (A*: the excited state). Reprinted with permission from ref. [86]. Copyright 2007 Elsevier.

With regard to the luminous form of the sensing layer, the intensity and stability of the optical signal are the core of the optical biosensor, which can be classified into passive type, photoluminescence type, and electroluminescence (ECL) type according to the way of generating the optical signal from the sensing layer. The working principles for the three types are shown in Figure 4 [86].

#### 3.1.1. Passive Optical Biosensor

In the absence of any other energy excitation conditions, the analyzed substance is combined with the bio-recognition membrane to generate the optical radiation by the chemical reaction itself for passive optical biosensors. There are two primary kinds of luminescence systems: one is chemiluminescence, which is a light phenomenon generated by chemical reactions. The other is bioluminescence, which is the phenomenon of luminescence that occurs within the body [87].

Studies on the theory of chemiluminescence and bioluminescence have recently made progress, and sensors based on these luminescent materials have also received increasing attention [88,89,90]. However, the passive optical biosensors for lactate detection have not been reported in recent years. Only in 2014 was a wearable chemiluminescent biosensor based on LOx/luminol/H_2_O_2_/HRP designed to monitor the athlete’s endurance performance by non-invasively detecting lactate in saliva and sweat. When the detection sample was dropped on the sensor, the analyte was measured quantitatively through the enzyme-catalyzed chemiluminescence reaction. The light emission was captured by the backside-illumination complementary metal-oxide semiconductor (BI-CMOS) sensors integrated into smartphones. The results showed that the detection limits of the chemiluminescence biosensor for lactate in sweat and saliva were 0.1 mmol/L and 0.5 mmol/L, respectively [91].

#### 3.1.2. Photoluminescent Optical Biosensor

An external excitation light source is required for a photoluminescent optical biosensor. The fixed excitation light source not only has a rather stable optical signal but also allows for wavelength adjustment. It can be classified into fluorescent labeling and unlabeling types depending on the varied structures of the biometric layer of the sensor. For fluorescent labeling type, the light source must deliver a sufficient amount of excitation power in the absorption band of the fluorescent labeling materials, and its energy is higher, so the wavelength is shorter than the emission peak of labeling materials. At present, most fluorescent labeling materials have been developed to emit in the visible spectrum. Therefore, the light source emitting in the blue to ultraviolet portion of the spectrum is suitable for many fluorescent labeling materials [92]. The unlabeling type uses the primary light (e.g., reflected light or scattered light) as the detection signal generated by the incident light passing through the sensing layer. The excitation source is usually evanescent wave, which can detect in a very small range. Photoluminescence does not involve the photochemical reaction process, unlike the passive type mentioned above. Hence, interference is quite minimal. Therefore, the stability and repeatability of the test are significantly improved [93].

Fluorescent labeling biosensors could reflect the content of fluorescent labeling materials ultimately bound to the biometric layer by detecting variations in fluorescence intensity [94,95]. Some researchers combined AIE-active fluorophore (TPE-HPro luminophore) with LOx to measure lactate. This method had good sensitivity and anti-interference properties [96]. A ratiometric fluorescent sensing membrane was also prepared by mixing LOx with Pt meso-tetra (pentafluorophenyl) porphyrin (PtP) doped in polystyrene particles and coumarin 6 (C6) in silica particles. In the oxidation reaction of lactate, the response of the sensing membrane at different lactate concentrations was measured by a multifunctional fluorescence microtiter plate reader at 635 nm wavelength. The results showed that the linear detection range of lactate was 0.1–0.8 mM, and the detection limit was 0.06 mM [97]. 

**Figure 5:**
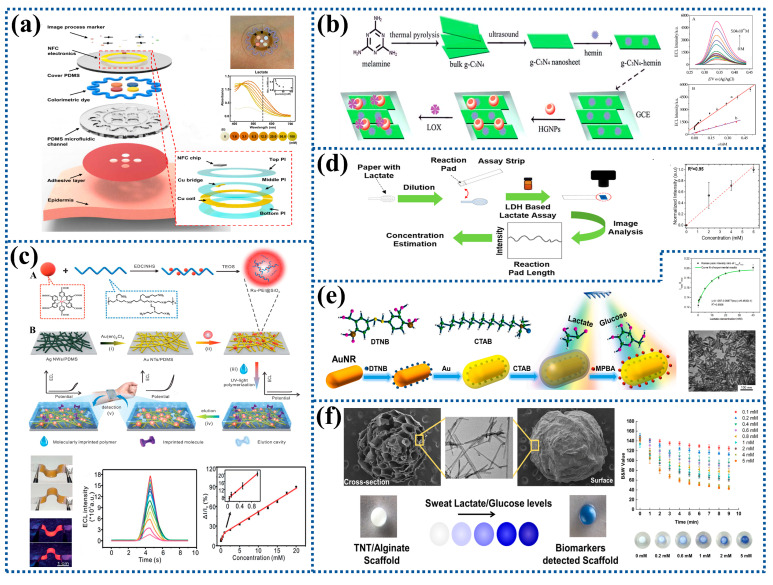
Preparation process and characterization of optical biosensors for lactate detection: (**a**) A soft, flexible, and stretchable microfluidic system for colorimetric analysis of lactate concentration. Reprinted with permission from ref. [102]. Copyright 2016 American Association for the Advancement of Science. (**b**) An electrogenerated chemiluminescent biosensor based on a g-C_3_N_4_-hemin nanocomposite and HGNPs for the detection of lactate. (A) ECL responses of the biosensor to lactate with different concentrations. (B) The curve of the linear relationship between ECL signal intensity and the concentration of lactate. Reprinted with permission from ref. [105]. Copyright 2014 Royal Society of Chemistry. (**c**) A flexible MIP-ECL sensor for epidermal analyte detection. (A) The synthesis of Ru-PEI@SiO_2_. (B) The fabrication of a flexible MIP-ECL sensor. (i) Galvanic conversion of Ag NWs/PDMS to an Au NT/PDMS electrode. (ii) Immobilization of HLNs on an Au NT electrode. (iii) UV-vis light-induced polymerization to form a target-imprinted MIP layer on HLNs/Au NTs. (iv) Elution of flexible MIP-ECL sensors. (v) Epidermal analyte detection. Reprinted with permission from ref. [106]. Copyright 2019 Royal Society of Chemistry. (**d**) A wearable permeable sweat sampling patch for sweat lactate detection. Reprinted with permission from ref. [109]. Copyright 2021 American Chemical Society. (**e**) A textile-based microfluidic device integrated SERS technology and colorimetric assay as a multifunctional sweat sensor. Reprinted with permission from ref. [113]. Copyright 2022 Elsevier. (**f**) A novel 3D titania dioxide nanotube/alginate hydrogel scaffold used to detect lactate in sweat. Reprinted with permission from ref. [114]. Copyright 2021 American Chemical Society.

In contrast to fluorescent labeling biosensors, the unlabeling ones do not require any additional fluorescent markers. Among the numerous unlabeling optical biosensors, colorimetric biosensors are particularly prominent [98,99,100,101]. For instance, a paper-based microfluidic system was prepared for the detection of lactate concentration in sweat based on color change in the chromogenic reagent caused by an enzymatic reaction of LDH and diaphorase between lactate and NAD^+^. The results showed that the color would change when the lactate concentration was within 1.5–100 mM, as shown in Figure 5a [102]. Alginate combined with LOx, HRP, and tetramethylbenzidine (TMB) could also be used to prepare a sensor for the rapid and reliable detection of lactate with the detection limit and the detection linear range of 6.4 mM and 10–100 mM [103].

#### 3.1.3. Electroluminescent Optical Biosensor

Optical sensors based on ECL are called electroluminescent optical biosensors. This type of sensor has a biometric layer as the working electrode, which is generally made of graphite, gold, or carbon. An electrochemical reaction will take place on the surface of working electrode leading to the release of optical signals that can be detected when a certain voltage is applied. Electroluminescent biosensors have numerous advantages over traditional biological and chemiluminescent sensors: high stability, high signal intensity, high detection sensitivity, recyclability, and control of reaction process and rate through voltage [104]. 

It was reported that g-C_3_N_4_-hemin nanocomposites were used to modify a GCE and hollow gold NPs (HGNPs) then self-assembled onto the electrode to adsorb LOx. As seen in Figure 5b, the prepared biosensor showed a good response performance to lactate with the linear detection range and the detection limit of 1.7 × 10^−8^–5.0 × 10^−4^ mM and 5.5 × 10^−9^ mM [105]. In addition, highly luminescent nanospheres (HLNs) were immobilized on Au nanotube (Au NT) networks, and then covered with elastic MIPs to fabricate a flexible ECL sensor. The results showed that the detection limit was 16.7 mM, the linear detection ranges were 50 μM–1.0 mM and 2.5–20.0 mM, and the sensor also demonstrated desirable fidelity, reusability, and high stability against disturbance, as shown in Figure 5c [106].

### 3.2. Preparation of Optical Biosensors

#### 3.2.1. Screen Printing

Screen printing is frequently used to create electrodes for optical biosensors because of its low cost, stability, and suitability for large-scale production. One research group prepared a ECL cloth-based biosensor based on luminol/H_2_O_2_ by screen printing, which can be used to detect lactate in saliva. The results showed that the detection limit was 0.035 mM and the detection range was 0.05–2.5 mM [5]. Another researcher also prepared an electrochromic biosensor through screen printing, including LOx and OS-polymer based anode connected to the PB cathode printed over a transparent poly(3,4-ethylenedioxythiophene) polystyrene sulfonate (PEDOT:PSS). The cathode display was effectively separated from the biosensing anode and shielded from the sample by an ion-gel made of Poly (vinylidene fluoride-co-hexafluoropropylene) (PVDF-co-HFP), a gelling agent, and ionic liquid 1-Ethyl-3-methylimidazolium trifluoromethanesulfonate (EMIM-Tf). Although its cathode was electrochromic, PEDOT:PSS achieved a transmittance of more than 90% and did not mask the color change of PB. The sensor exhibited lactate concentrations ranging from 0 to 10 mM on the electrochromic display [107].

#### 3.2.2. Immobilization

The immobilization of biological functional substances, such as enzymes, antigens, or antibodies, on the surface of the carrier is often achieved via embedding, adsorption, covalent bonding, and cross-linking [108]. LOx or LDH is often mixed with cellulose, dyes, and other enzyme solutions and immobilized on the carrier surface, as shown in Figure 5d [109,110,111]. In a study, a mixture of carbon nanotube suspension and potassium ferrocyanide solution was firstly dropped onto luminol surface, and then loaded by a combination of LDH, nicotinamide adenine dinucleotide (NAD), and pyruvate oxidase (PYOD) to build an electroluminescent lactate biosensor with the detection limit of 8.9 × 10^−12^ mol L^−1^ [112].

#### 3.2.3. Others

A textile-based multifunctional sweat sensor was developed by combining surface-enhanced Raman scattering (SERS) technology and colorimetric analysis technology. Core-shell gold nanorods (Au NRs) were used to construct sandwiched Raman reporters, which were then applied as SERS tags to detect lactate in sweat. As can be seen in Figure 5e, it had the detection range of 0.1–40 mM and the detection limit of 0.05 mM [113]. Other researchers created a novel 3D titanium dioxide nanotube (TNT)/sodium alginate hydrogel scaffold for detecting lactate in artificial sweat with the detection limit of 0.069 mM. The color change of TNT/alginate scaffolds at high lactate concentrations (10–100 mM) is depicted in Figure 5f [114].

In Table 2, different types of optical biosensors are compared mainly in terms of the electrode materials, detection methods, detection objects, and various properties of the sensor. Through comparison, it is found that enzyme-based optical biosensors have lower detection limits than non-enzymatic types, but non-enzymatic optical biosensors are more stable.

## 4. Semiconductor Biosensors

### 4.1. Field-Effect Transistors

**Figure 6:**
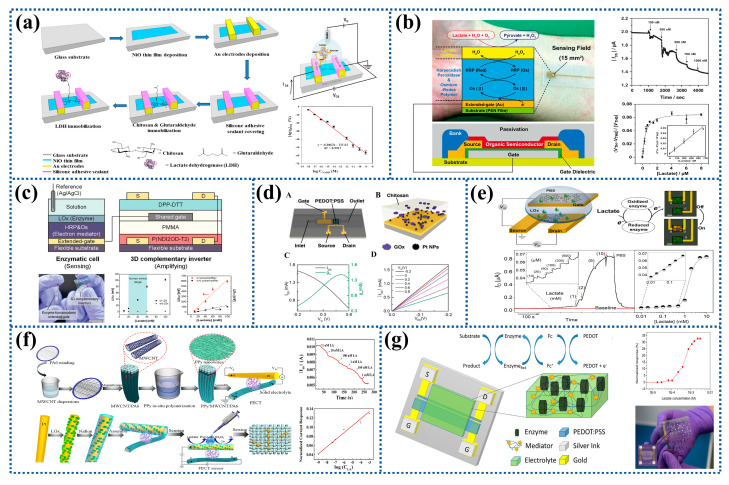
Preparation process and characterization of semiconductor biosensors for lactate detection: (**a**) Nickel oxide thin-film field-effect transistor based on radio frequency. Reprinted with permission from ref. [117]. Copyright 2017 Elsevier. (**b**) An extended-gate type OFET for lactate detection in aqueous media. Reprinted with permission from ref. [121]. Copyright 2015 Elsevier. (**c**) An organic voltage amplifier for lactate sensor on flexible plastic foil. Reprinted with permission from ref. [123]. Copyright 2020 WILEY-VCH. (**d**) OECT used as highly sensitive lactate sensors by modifying the gate electrode with LOx and poly(n-vinyl-2-pyrrolidone) -capped Pt NPs. (A) Schematic diagram of lactate sensor based on OECT integrated with microfluidic channel (LOx solution was used instead of GOx solution). (B) Gate electrode modification of device. (C) Transfer curve and corresponding transconductance curve of an OECT. (D) Output curve of OECT. Reprinted with permission from ref. [128]. Copyright 2016 WILEY-VCH. (**e**) A cumulative mode OECT prepared using n-type polymers. Reprinted with permission from ref. [131]. Copyright 2018 American Association for the Advancement of Science. (**f**) FECTs based on multi-walled carbon nanotube and PPy composites for noninvasive lactate sensing. Reprinted with permission from ref. [132]. Copyright 2020 Springer. (**g**) An organically modified sol-gel solid electrolyte for printed OECT-based lactate biosensor. Reprinted with permission from ref. [134]. Copyright 2015 Springer.

Field-effect transistors (FETs) are widely used in the detection of various biomolecules due to their high sensitivity, fast analysis, label-free, small size, and simple operation. In contrast to conventional electrochemical biosensors, FETs can be miniaturized to the micro or nanoscale, thus enabling detection at the molecular level and integration into small chips [116]. In recent years, metal oxide (e.g., CuO, ZnO, NiO, and TiO_2_) nanostructures have been used to improve sensor performance due to their advantages such as high specific surface area and excellent electrocatalytic efficiency. For example, LDH could be immobilized on the surface of radiofrequency-sputtered nanostructured NiO films modified by CS to develop a FETs-based lactate biosensor, as shown in Figure 6a. The biosensor exhibited efficient sensing response characteristics with the linear dynamic range and the detection limit of 1 aM–1 pM and 0.5 aM [117]. CNTs can be used as active semiconductor materials for FETs due to their excellent properties including high specific surface area, good biocompatibility, electrochemical stability, and good electrical conductivity. The results showed that the sensitivity of the sensor was 2.198 μA/Decade, and it still had a good mechanical deformation ability after three weeks, showing good mechanical stability, but its sensitivity was reduced [118].

In recent years, various properties of organic materials have been continuously exploited and played an increasingly important role in the electronics industry. Based on the advantages of organic materials, such as low cost, easy performance adjustment, and simple processing, the introduction of them into FETs is expected to produce inexpensive or flexible organic field-effect transistors (OFETs) [119]. For example, Minami’s group [120] prepared low-cost ultra-flexible OFETs for the detection of lactate and used them as disposable sensors to achieve non-invasive health monitoring. The gate electrode of OFETs was modified with LOx and HRP-osmium redox polymer on flexible plastic film substrates to achieve the detection limit of 66 nM, and the detection range of 100–1000 nM, as shown in Figure 6b [121]. In addition, the OFETs were also developed on glass plate substrates with the detection range of 0–10 mM, which expanded the detection range of lactate compared with previous work [122]. To further enhance the sensitivity of the sensor, an organic voltage amplifier based on 3D stacked organic complementary transistors fabricated by vertically stacking P-type OFECTs (POFETs) on N-type OFECTs (NOFETs) and sharing a gate electrode between them was proposed to amplify the detection signal, as shown in Figure 6c. Due to high amplification, the sensing device could detect lactate concentration in the human sweat range of 20–60 mM with the high sensitivity of 6.82 mV mM^−1^ [123].

### 4.2. Organic Electrochemical Transistors

Organic electrochemical transistors (OECTs) invented by White in the mid-1980s [124], have the advantages of flexible design, low operating voltage, high input impedance and good stability. They have dual functions of sensing and amplification, and can be used to biomolecule detection, environment monitoring, and medical diagnosis. OECTs can be fabricated by inkjet printing, flexographic printing, or screen printing and are very suitable for application in biosensing.

Most of the studies on OECTs are based on P-type semiconductor materials, such as PPy, PANI, and PEDOT:PSS, because their preparation process is relatively simple and low-cost. Among them, PEDOT:PSS is the most widely studied [125,126]. For instance, PEDOT:PSS and PVA were mixed and deposited on the gate electrode, and then CS-FC/LOx mixture was drop-coated on the surface of PEDOT:PSS/PVA to prepare OECTs with the detection limit of 50 μM and the detection range of 0.1–2 × 10^−3^ M [18]. In order to improve the sensitivity of the sensor, the electrode could be modified with metal Ns [127]. For example, a researcher successively deposited Pt NPs and mixed solution of LOx and CS on PEDOT:PSS substrate. The improved OECTs had a high sensitivity, and the detection limit of lactate could be reduced to 10^−6^ M, as shown in Figure 6d [128].

OECTs based on N-type organic semiconductor materials have relatively high signal on/off response and sensitivity [129]. In 2016, a N-type organic semiconductor material (conjugated polymer (naphthalene-1,4,5,8-tetracarboxylic diimide (NDI)) was firstly used to prepare OECTs for detecting lactate content with a high sensitivity of 10 μA μM^−1^ and a wide concentration range of 10 μM–10 mM over a physiologically relevant concentration range, and a very fast response of about 2 S to low metabolite concentrations (<0.3 mM) in the linear detection range [130]. To further optimize the lactate sensor, a fully integrated, miniaturized, and easily fabricated transistor device was fabricated. Au contacts (located at the source, drain, and gate) and interconnects were patterned on the glass substrate. Then, the NDI repeat units and an electron-rich unsubstituted bithiophene repeat unit (T_2_) was spun-cast on the gate electrode. The results showed that the detection range was from 10 μM to 10 mM, as shown in Figure 6e [131].

In recent years, smart wearable devices have become a development trend in electronic products. Fiber-based organic electrochemical transistors (FECTs) possessing many advantages, including small size, woven construction, lightweight, and good flexibility, have been developed. Therefore, FECTs have broad application prospects in the fields of wearable flexible electronics and biosensing. Some scholars coated MWCNTs on nylon 6 (PA6) fibers, and then applied a layer of PPy nanowires through in-situ polymerization. The results demonstrated that the response time was 0.6–0.8 S and the linear response range was 1 nM–1 mM, as shown in Figure 6f [132].

The practical application of the majority of the present OECTs has been constrained because they employ aqueous electrolytes. In order to make them into wearable devices, the electrolytes need to be solid. A flexible transistor-based biosensor with a solid-state electrolyte was first demonstrated in 2012 [133]. Subsequently, an organically modified sol-gel electrolyte was applied to an OECT and used to detect lactate in human sweat with the detection range of 0.1–2.3 mM, as shown in Figure 6g [134].

## 5. Self-Powered Biosensors

At present, batteries are generally required as a power supply for the majority of sensors. Due to the inconvenience of charging and replacement, the batteries or other power units embedded in the sensor system limit their long-term and real-time use. Therefore, the development of a wearable sensing system with a self-powered function is particularly important for daily use.

### 5.1. Piezoelectric Biosensors

In recent years, piezoelectric biosensing technology has been introduced as a new tool for monitoring sweat metabolites. At present, the commonly used piezoelectric materials can be roughly divided into two categories: One is inorganic piezoelectric materials, such as lead zirconium titanate (PZT), ZnO, and barium titanate (BaTiO_3_). The other is organic piezoelectric materials, including polyvinylidene fluoride (PVDF) and its copolymer polyvinylidene fluoride-trifluoroethylene (PVDF-TrFE), etc.

**Figure 7:**
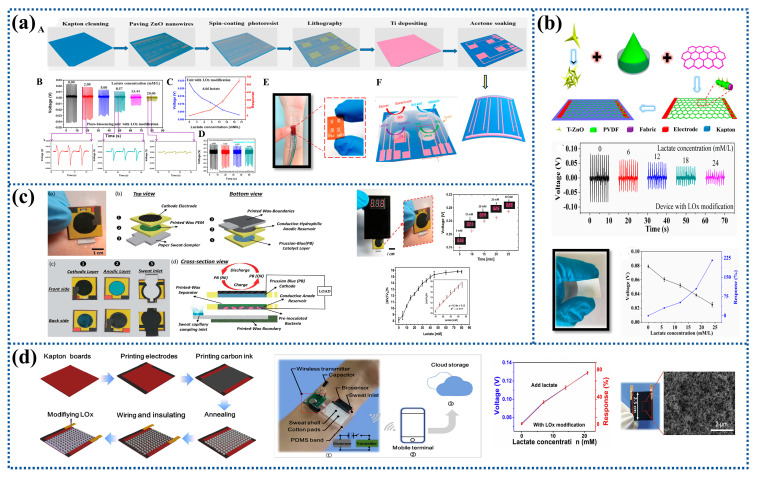
Preparation process and characterization of self-powered biosensors for lactate detection: (**a**) A self-powered piezoelectric biosensor based on enzyme/ZnO nanoarrays. (A) The fabrication process of the electronic skin. (B) The piezoelectric impulse of the piezoelectric biosensor. (C) The piezoelectric output voltage and response of the piezoelectric biosensor in different concentration of lactate. (D) The detection limit and the resolution of the piezoelectric lactate biosensor. (E) Optical images of the electronic skin. (F) The electronic skin for detecting lactate. Reprinted with permission from ref. [135]. Copyright 2017 American Chemical Society. (**b**) A self-powered piezoelectric biosensing textiles based on PVDF/T-ZnO. Reprinted with permission from ref. [136]. Copyright 2019 MDPI. (**c**) MFC as a self-powered lactate sensor that can be used to monitor sweat lactate. Reprinted with permission from ref. [144]. Copyright 2019 IEEE Proceedings. (**d**) A self-powered lactate biosensor fabricated from porous carbon film (modified with LOx). Reprinted with permission from ref. [145]. Copyright 2019 Elsevier.

The piezoelectric materials are often combined with enzymes to prepare piezoelectric lactate biosensors. For instance, ZnO nanowires were modified with LOx to prepare a piezoelectric biosensing unit matrix, which could be used as a self-powered wearable non-invasive electronic skin for detecting the lactate content in sweat with the detection limit of about 0.10 mM L^−1^ as shown in Figure 7a. Its working principle was the reaction coupling effect of the enzyme and ZnO nanowires. The piezoelectric output of the piezoelectric biosensing unit could be considered as power supply and biosensing data and depended on the analyte concentration in sweat [135]. It could also be achieved by a simple wet-chemical method where a PVDF binder was used to firmly fix the tetrapod-shaped ZnO (T-ZnO) nanowires to the textile and then LOx was slowly dropped onto their surface, as shown in Figure 7b. The output voltage of the LOx-modified device decreased with the increase of lactate concentration, so the device allowed real-time monitoring of the lactate concentration in the sweat of athletes [19,136].

However, the wearable piezoelectric biosensor is still a new self-powered proof-of-concept device, and its accuracy and time of use need to be rigorously evaluated in practical applications.

### 5.2. Fuel Cell-Based Biosensors

Fuel cells can directly convert the chemical energy existing in the fuel into electrical energy, but most materials of fuel cell are scarce and expensive. To solve this problem, enzymes extracted from glucose oxidase, laccase, and bilirubin oxidase are often used as catalysts to replace precious metals to prepare enzymatic biofuel cell (EBFC), which can convert biochemical energy into electrical energy through an enzymatic electrochemical reaction [137,138].

A research group prepared an integrated self-powered chemical biosensing system with a digital wireless readout function using an EBFC-powered nano-CMOS chip for the first time, which successfully detected changes in lactate concentration between 2.5 and 15 mM [139]. The EBFC could also directly use the concentration change of the fuel to generate different voltages, thereby indirectly responding to the concentration of the analyte. Another research group prepared a self-powered amperometric lactate biosensor, in which dimethyl FC-modified linear poly(ethyleneimine) (FcMe_2_-LPEI) hydrogel was used as the anode, and the enzyme as a cathode. LOx was immobilized on both the cathode and anode. The self-powered biosensor had a detection range of 0–5 mM and a sensitivity of 45 ± 6 µA mM^−1^ cm^−2^ [140]. In addition, the anode and cathode could be modified with D-lactate dehydrogenase (D-LDH) and bilirubin oxidase (BOD) to promote the oxidation and reduction of lactate and molecular oxygen [141]. To make the sensor flexible, a stretchable self-powered biosensor was fabricated on the PDMS substrate to detect the concentration of lactate in sweat. The Au electrode was first sprayed with graphene suspension. Then, LOx, BSA, and glutaraldehyde were mixed and dropped onto the graphene coating to prepare the anode. Catalytic inks for biocathodes were prepared similarly using laccase (Lac) instead of oxidase. The results showed that the sensor had a lactate detection sensitivity of 2.48 mV mM^−1^ with excellent mechanical properties and stable output even under 30% stretch condition [142].

Due to the limited lifespan, environmental instability, and the requirement of expensive and time-consuming purification for enzymes, enzyme-based biosensors exhibit certain limitations. In contrast, bacterial cells have the advantages of low cost and good stability compared with enzymes and have the potential to be an alternative to enzymatic fuel for non-invasive and self-powered biomimetic sensing [143]. The biodegradable organic matrix lactate can be oxidized using electrogenic bacterial cells to generate electrons based on their electrochemical energy conversion. One research group integrated the anode, wax-based proton exchange membrane (PEM), and PB-cathode into three functional paper layers to prepare microbial fuel cell (MFC). The results showed that the output voltage was linear in the range of 0.0–40.0 mM, as shown in Figure 7c [144].

### 5.3. Others

A novel self-powered wearable sweat lactate biosensor was established by coupling sweat evaporation power generation with the biosensing process. The self-powered biosensor was constructed from a porous carbon membrane modified with LOx and could generate electricity through natural sweat evaporation. The output voltage depended on sweat lactate concentration, increasing with the increase of lactate concentration in sweat, as shown in Figure 7d [145].

## 6. Flexible Substrate Materials for Wearable Biosensors

In recent years, there has been an increasing demand for wearable biosensing devices for detecting lactate. Soft, thin, and non-irritating substrate materials ought to be chosen because they are in direct contact with the skin. PDMS, PET, paper, fabric, hydrogel, and PI are currently the most widely utilized flexible substrate materials due to their advantages of soft texture, light weight, stable chemical properties, low production cost, and good biocompatibility [100,146,147,148].

### 6.1. PDMS

**Figure 8:**
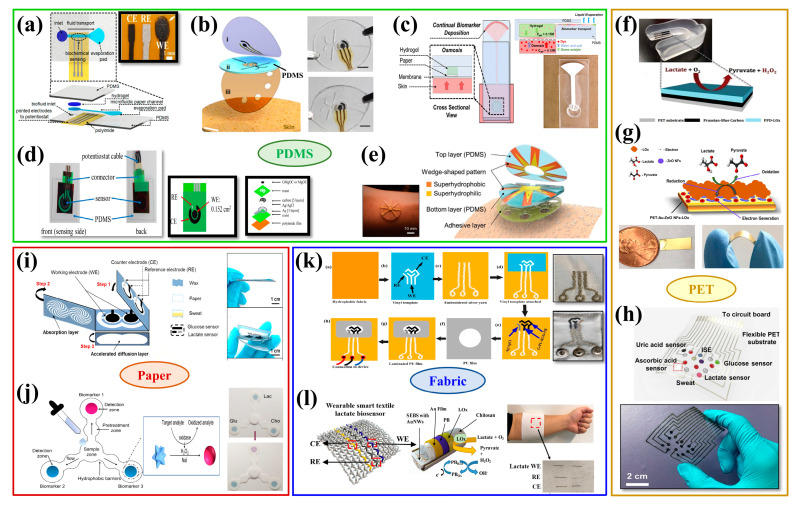
Biosensors for lactate detection based on different substrate materials: (**a**–**e**) Biosensors for detecting lactate based on PDMS. Reprinted with permission from ref. [55,109,149,150,152]. Copyright 2019 IEEE Proceedings. Copyright 2017 American Chemical Society. Copyright 2021 American Chemical Society. Copyright 2021 Elsevier. Copyright 2021 Wiley-VCH. (**f**–**h**) Biosensors for detecting lactate based on PET. Reprinted with permission from ref. [38,75,153]. Copyright 2014 Royal Society of Chemistry. Copyright 2020 IEEE Sensor Journal. Copyright 2019 Advancement of Science. (**i**,**j**) Biosensors for detecting lactate based on paper. Reprinted with permission from ref. [30,155]. Copyright 2021 Elsevier. Copyright 2021 MDPI. (**k**,**l**) Biosensors for detecting lactate based on fabric. Reprinted with permission from ref. [76,157]. Copyright 2021 Elsevier. Copyright 2022 Elsevier.

Among many flexible substrate materials, PDMS is widely favored by researchers due to its easy availability, low elastic modulus, and large tensile properties. A PDMS-based wearable lactate monitoring sensor system could achieve continuous, non-invasive monitoring of lactate at low sweating rates, as shown in Figure 8a [149]. In another study, an epidermal microfluidic device was fabricated using two soft PDMS layers and a double-sided sticky layer. As seen in Figure 8b, the first PDMS layer integrated the electrode system, and the second layer contained microfluidic channels and sweat storage regions. The flexible microchip device could easily adhere to the epidermis and formed conformal contact with skin sweat pores, allowing the rapid flow of sweat to the stored areas, while being able to withstand repeated mechanical deformation by the wearer [150].

In addition to developing biosensors that can detect lactate stably and accurately, it is also necessary to consider how to effectively collect detection substances and deliver to the sensors for detection [151]. As illustrated in Figure 8c, PDMS and hydrogel were combined to create a non-invasive sweat sampling patch that could constantly measure changes in lactate concentration in sweat over a longer period of time [109]. Other researcher developed a lactate sensing system with a microfluidic sweat collector made of PDMS. As demonstrated in Figure 8d, the lactate sensing device was portable, convenient to wear on the skin, and capable of continuously monitoring the lactate content in sweat [55]. Furthermore, a patch with the functions of directional transport and high-efficiency sweat collection was found to be able to rapidly and continuously monitor the lactate in sweat. Cactus spine-inspired wedge-shaped wetting patterned channels were used on a layered micro/nanostructured surface, followed by encapsulation of the channels with PDMS to prepare the patch, as shown in Figure 8e [152].

### 6.2. PET

PET not only has excellent mechanical properties and insulation, but also has good temperature resistance and corrosion resistance, and is often considered as an ideal substrate material for a flexible sensor [148].

Some researchers prepared a lactate biosensor by using PET as the base material and then integrated it into an easily detachable wearable mouthguard platform. The wearable biosensor was capable of the non-invasive, highly sensitive, and stable detection of lactate concentration in saliva, as shown in Figure 8f [38]. A highly sensitive, selective, and flexible enzyme sensor based on ZnO nanoflakes (ZnO-NFs) for the non-invasive detection of lactate in sweat was also prepared with a single-step sonochemical process where gold was plated on flexible PET and used as a substrate for the sensor, as shown in Figure 8g [153]. In order to integrate the sensor with signal acquisition and data transmission to prepare a sensor array, digital laser processing technique could be used to directly print the circuits onto a flexible PET substrate to prepare a wearable patch, as shown in Figure 8h [75].

### 6.3. Paper

Paper is one of the most commonly used flexible substrate materials for wearable biosensors, and its main component is cellulose. Because the natural capillary absorption of paper is conducive to sample collection, paper-based biosensors can be well utilized in medical diagnosis and other applications [154].

One research group prepared a low-cost, self-contained, and highly integrated sensing paper (HIS paper) that could detect the concentration of lactate in sweat in real-time. The hydrophobic protective wax, conductive electrodes, and MXene/methylene blue active components were assembled into the HIS paper using a simple printing process, as shown in Figure 8i [30]. The majority of optical biosensors are used for qualitative lactate concentration detection. A paper-based colorimetric sensing device was fabricated to detect glucose, cholesterol, and lactate in saliva, as shown in Figure 8j. By dropping a saliva sample into the central area of the device, the sample was analyzed in the detection area using a controlled reaction [155]. Another research group also prepared a paper-based colorimetric wearable biosensor, which could simultaneously measure the concentration and volume of lactate in sweat [115].

### 6.4. Fabric

Fabric is one of the most common materials that come into contact with the human body and is composed of flexible materials such as natural or synthetic fibers. Compared with wearable biosensors based on other substrate materials, fabric-based ones have the advantages of flexibility, moisture absorption, and breathability and can meet the requirements of 3D distortion of the human body to achieve the maximum fit between the device and human body [156].

There are two major methods for creating flexible fabric-based wearable sensors. The first method can integrate a sensor on a commercial fabric or print functional materials onto the fabric to form a sensor with specific functions. However, the high roughness, porosity, and moisture-wicking qualities of the fabric may cause the functional materials to separate from the fabric and affect the performance of the sensor. Therefore, some scholars prepared a three-electrode templates on a hydrophobic fabric substrate. The conductive silver fibers were first stitched into the fabric substrate. Then, the modified graphene-based nanocomposites were filled into the blank areas to be used as the working electrode and the counter electrode, and Ag/AgCl was used as the reference electrode, as shown in Figure 8k. The sensor could still detect the lactate concentration after washing in a domestic washing machine, showing good reproducibility [157]. The second method is to fabricate wearable sensors by weaving the fabricated yarn electrodes into fabrics. For example, a three-electrode system was fabricated by using carbon-coated nylon wire as the working electrode and counter electrode and Ag/AgCl-coated nylon wire as the reference electrode. The prepared yarn electrodes were then woven and conducted hydrophobic treatment to prevent sweat from penetrating into the fibers. The results showed that the sensor exhibited a good linearity over the concentration range of 4–20mM and a good robustness with small variations of output signal, demonstrating the potential applicability of the wearable sensor [68]. In addition, a wearable lactate biosensor based on gold fiber electrodes was fabricated. Gold fibers, PB/LOx/CS-coated gold fibers, and Ag/AgCl-coated gold fibers were used as counter electrode, working electrode, and reference electrode, respectively, and the electrodes were then woven into textiles, as shown in Figure 8l. The results demonstrated that the sensitivity was 14.6 μA mM^−1^ cm^−2^ and remained unchanged under tensile strain up to 100% [76].

## 7. Conclusions

In this paper, non-invasive wearable lactate biosensors based on flexible, biocompatible, and comfortable substrates for lactate monitoring are reviewed. In general, most of the prepared lactate biosensors are electrochemical biosensors due to their convenient preparation and more accurate detection of lactate content, and they are suitable for patients who need to know the lactate content accurately. Semiconductor biosensors have also been widely studied in recent years due to the growth of the semiconductor industry and can be used to detect lactate at low concentrations due to their dual functions of amplification and sensing. Compared with other types of biosensors, optical biosensors are less accurate in detecting lactate content. A self-powered lactate biosensor is also studied to be more suitable for body wear. However, the cost is relatively high, and its accuracy and usage time still need to be rigorously evaluated in practical applications.

Lactate biosensors can be roughly divided into enzymatic biosensors and non-enzymatic biosensors. For enzyme-based biosensors, enzyme activity is easily affected by external factors, such as temperature and pH value, and may be degraded under improper storage and use, which generally results in poor reproducibility of test results and significant data errors, thus affecting the sensitivity of enzyme-based biosensors to lactate as well as their capacity for long-term monitoring. In contrast, non-enzymatic biosensors can offer superior stability and are less susceptible to external influences, but their detection accuracy is limited. Therefore, it is crucial to prepare a non-enzymatic biosensor with a high detection accuracy. There are several challenges in the current research of lactate biosensors, such as evaporation or accumulation of biological fluid samples without penetrating the epidermis and contamination of the samples by impurities on the skin surface. The level of integration between biosensors and intelligent wearables is not high, and further study is still needed to determine how to combine biosensing technology with intelligent wearables. It is expected that multi-functional and stable wearable biosensors can be prepared to provide health protection for people’s daily life in the future.

## Figures and Tables

**Table 1:** Comparison of characteristics for various electrochemical lactate biosensors.

Working Electrodes	Measurement Techniques	Enzymes	Sensing Fluids	Sensitivity	Linearity	Detection Limits	Ref.
CNT/TTF/LOx/CS	Amperometry	LOx	Sweat	14.66 µA mM^−1^ cm^−2^	1–20 mM	—	[14]
PB/SPE/LOx/Nafion	Amperometry	LOx	Saliva	—	0.025–0.25 mM	0.01 mM	[9]
PB/BSA/LOx/PVC	Amperometry	LOx	Sweat	96 nA mM^−1^	0–28 mM	—	[74]
LOx–Cu-MOF/CS/Pt/SPCE	Amperometry	LOx	Sweat, Saliva	14.650 µA mM^−1^	0.00075–1.0 mM	0.75 µM	[60]
Pt/SilkNCT/LOx/CS	Amperometry	LOx	Sweat	174.0 nA mM^−1^	5–35 mM	0.5 mM	[75]
LOx/CNTs/Ti_3_C_2_T_x_/PB/CFMs	Amperometry	LOx	Sweat	11.4 µA mM^−1^ cm^−2^	0–20 mM	0.67 μM	[31]
Au/TTF/CNT/LOx/CS/PVC Au/TTF/CNT/LOx/CS	Amperometry	LOx	Sweat	3.28 ± 8 μA mM^−1^ 0.43 ± 0.11 μA mM^−1^	0–20 mM 0–30 mM	—	[45]
PB/LOx/CS/Au	Amperometry	LOx	Sweat	14.6 μA mM^−1^ cm^−2^	0–30 mM	—	[76]
Cabon/PB/LOx/ PVC/DOS/ETH500	Amperometry	LOx	Sweat	−9.4 nA mM^−1^	1–50 mM	0.11 mM	[77]
SPE/PB/LOx + GO-Ch	Amperometry	LOx	Sweat	0.39 μA mM^−1^ cm^−2^	1.0–50.0 mM	—	[58]
LOx/BSA/PEGDE/AuNNs/Au	Amperometry	LOx	Sweat	—	5–25 mM	54 μM	[16]
CNTs/CNT-PB/CS/LOx	Amperometry	LOx	Sweat	—	0.25–35 mM	0.25 mM	[78]
LSG/Pt/CS/LOx	Amperometry	LOx	Saliva	35.8 µA mM^−1^ cm^−2^	0.2–3 mM	0.11 mM	[73]
PB/LOx/CS/carbon	Amperometry	LOx	Sweat	0.027 ± 0.002 µA mM^−1^	5–30 mM	—	[41]
carbon/PB/LOX/ graphene/Nafion	Amperometry	LOx	Sweat	10 µA mM^−1^ cm^−2^	0–20 mM	350 nM	[79]
PB/rGO/Au/LOx	Amperometry	LOx	Sweat	40.6 μA mM^−1^ cm^−2^ 1.9 μA mM^−1^ cm^−2^	1–222 μM 0.222–25 mM	—	[65]
LOD/BSA/GA/AgNP/Nafion	Amperometry	LOD	Sweat	262 nA mM^−1^ cm^−2^	1–25 mM	—	[80]
Ag/AgCl/Carbon graphite/LOD	Amperometry	LOD	Sweat	—	0.1–1 mM	84.8 µM	[81]
Carbon/OS polymer/ LOD	Amperometry	LOD	Sweat	376.5 nA mM^−1^	25–1000 µM	—	[56]
LOD/BSA/FC/GA/Nafion	Amperometry	LOD	Saliva	21.8 µA mM^−1^ cm^−2^	0–2000 μM	—	[82]
NiCo-LDH/SPCE	Amperometry	LDH	Sweat	83.98 μA mM^−1^ cm^−2^	2–26 mM	0.4 mM	[63]
BP/Polmethylene green/LDH	Amperometry	LDH	Sweat	0.2 μA mM^−1^	5–100 mM	—	[83]
Carbon paper/Cu-catecholates	Amperometry	—	Sweat	0.11 mA mM^−1^ cm^−2^	0.02–21.35 mM	10 μM	[34]
MWCNT/PPy	Amperometry	—	Sweat	2.9 µA mM^−1^ cm^−2^	—	51 µM	[7]
NiS-NC@NiS-MS	Amperometry	—	Urine	2.2 μA μM^−1^ cm^−2^	0.5–88.5 μM	0.5 μM	[36]
HS-NiS	Amperometry	—	Urine	0.655 μA μM^−1^ cm^−2^	0.5–88.5 μM	0.023 μM	[35]
MIP/Ag-Au NPs/SPCE	Amperometry	—	Sweat	0.88066 μA mM^−1^	1–220 μM	0.003 μM	[66]
PANI/SPCE/Nafion	Amperometry	—	Sweat	18.62 nA mM^−1^ 4.25 nA mM^−1^	0.25–10 mM 10–60 mM	0.083 mM	[61]
ZnO NWs/TTF/ LOx/CS/GA/Nafion	Potentiometric	LOx	Sweat	—	0–25 mM	3.61 mM	[25]
MIPs-AgNWs/Carbon	DPV	—	Sweat	—	10^−6^–0.1 M	0.22 μM	[67]
Poly(3-APBA)	EIS	—	Sweat	—	3–100 mM	1.5 mM	[10]
GO-LOD	EIS	LOD	Sweat	—	1–100 mM	1 mM	[47]
ZnO/LOx	EIS	LOx	Sweat	—	1–100 mM	1 mM	[54]
PEDOT/LOx/SPCE	EIS	LOx	Sweat	43.42 µA mM^−1^ cm^−2^ 0.32 µA mM^−1^ cm^−2^	0.25–1 mM 1–40 mM	0.083 mM	[84]

BSA: Bovine Serum Albumin, SilkNCT: Silk fabric–derived intrinsically Nitrogen (N)–doped carbon textile, CFMs: Carbon Fiber Membranes, DOS: bis(2-ethylhexyl) sebacate, ETH500: membrane containing tetradodecylammonium tetrakis(4-chlorophenyl) borate, Au NNs: Gold Nanopine Needles, OS: Osmium, FC: Ferrocene, NiS-NC: Nanoclusters of Nickel-Sulfides, NiS-Ms: NiS microsphere, HS-NiS: Hollow Sphere structured NiS.

**Table 2:** Comparison of characteristics for various optical lactate biosensors.

Support of Immobilization	Measurement Techniques	Enzymes	Sensing Fluids	Linearity	Detection Limit	Ref.
LOx/TPE-HPro	fluorescence	LOx	Saliva	0–200 μM	5.5 μM	[96]
fluorescein/Fe (III) complex	fluorescence	—	Sweat	1.0–12.5 mM	0.4 mM	[95]
LOx/HRP/TMB	Colorimetric	LOx	Sweat	10–30 mM	0.06 mM	[115]
LOx/HRP/4-aminoantipyrin/TOOS	Colorimetric	LOx	Sweat	0–25 mM	—	[17]
Alginate/TNT/LOx	Colorimetric	LOx	Sweat	10–100 mM	0.069 mM	[114]
LOD/HRP/TMB/ Alginate	Colorimetric	LOD	Artificial sweat	10–100 mM	6.4 mM	[103]
LDH/NAD^+^/formazan dyes	Colorimetric	LDH	Sweat	1.5–100 mM	—	[102]
Au NRs@DTNB@Au	Colorimetric	LDH	Sweat	0.1–40 mM	0.05 mM	[113]
MIP/Ru-PEI@SiO_2_/Au NTs	ECL	—	Sweat	0.05–1.0 mM 2.5–20.0 mM	16.7 mM	[106]
LOx/luminol	ECL	LOx	Saliva	0.05–2.5 mM	0.035 mM	[5]
NAD/PYOD/LDH/ luminol	ECL	LDH	Sweat	—	8.9 μM	[112]

TOOS: N-Ethyl-N-(2-hydroxy-3-sulfopropyl)-3-methylaniline, sodium salt, dihydrate, DTNB: 5,5′-dithiobis(2-nitrobenzoic).

## Data Availability

Not applicable.
